# Genetic responses of plants to urban environmental challenges

**DOI:** 10.1007/s00425-025-04678-1

**Published:** 2025-04-04

**Authors:** Angela Carfora, Francesca Lucibelli, Paola Di Lillo, Sarah Maria Mazzucchiello, Giuseppe Saccone, Marco Salvemini, Marianna Varone, Gennaro Volpe, Serena Aceto

**Affiliations:** Department of Biology, University of Naples Federico II, Via Cintia 26, 80126 Naples, Italy

**Keywords:** Urbanization; Urban stressors; Plant adaptation; Gene expression

## Abstract

**Main conclusion:**

This review aims to describe the main genetic adaptations of plants to abiotic and biotic stressors in urban landscapes through modulation of gene expression and genotypic changes.

**Abstract:**

Urbanization deeply impacts biodiversity through ecosystem alteration and habitat fragmentation, creating novel environmental challenges for plant species. Plants have evolved cellular, molecular, and biochemical strategies to cope with the diverse biotic and abiotic stresses associated with urbanization. However, many of these defense and resistance mechanisms remain poorly understood. Addressing these knowledge gaps is crucial for advancing our understanding of urban biodiversity and elucidating the ecological and evolutionary dynamics of species in urban landscapes. As sessile organisms, plants depend heavily on modifications in gene expression as a rapid and efficient strategy to survive urban stressors. At the same time, the urban environment pressures induced plant species to evolve genotypic adaptations that enhance their survival and growth in these contexts. This review explores the different genetic responses of plants to urbanization. We focus on key abiotic challenges, such as air pollution, elevated CO_2_ levels, heavy metal contamination, heat and drought stress, salinity, and biotic stresses caused by herbivorous insects. By examining these genetic mechanisms induced by urban stressors, we aim to analyze the molecular pathways and genetic patterns underlying the adaptation of plant species to urban environments. This knowledge is a valuable tool for enhancing the selection and propagation of adaptive traits in plant populations, supporting species conservation efforts, and promoting urban biodiversity.

## Urbanization and its impact on biodiversity

In recent years, urban and suburban areas have undergone rapid expansion. Today, most of the global population resides in cities, and urbanization continues to rise across all regions due to population growth and the progressive shift away from agricultural employment (Shochat et al. 2006; Johnson and Munshi-South 2017). The urbanization process, driven by cities, suburbs, and infrastructure development, leads to dramatic changes in natural ecosystems, making it a key concern in conservation biology. Within urban areas, vegetation is restructured, and species compositions are altered in abundance, richness, and evenness (Shochat et al. 2006; Grimm et al. 2008). Understanding the complex processes driving these changes in dynamic urban ecosystems and the mechanisms underlying species adaptation represents a significant challenge. Addressing this challenge requires a multidisciplinary approach involving biologists, anthropologists, sociologists, and geographers (Grimm et al. 2008). Collaboration between disciplines is a good strategy for designing sustainable urban spaces using a "One Health" approach that recognizes the interconnection between human, animal, and environmental health, emphasizing that the health of one influences that of others. This concept is crucial for promoting inclusive and sustainable urban spaces that enhance the well-being of the entire community (Bruno et al. 2024).

Cities are fully functioning ecosystems that shape biodiversity and drive its evolution in response to novel urban conditions (Bolund and Hunhammar 1999; McKinney 2006). Urban environments act as hotspots of evolution, promoting rapid ecological and evolutionary changes by creating new species interactions and altering ecological niches (Miles et al. 2019b; Gallo et al. 2019). Evolutionary processes triggered by urban environments include changes in allele frequencies, the emergence of new mutations, genetic drift, and gene flow (Verrelli et al. 2022; Alberti et al. 2020; Johnson and Munshi-South 2017). For instance, urban pollution can increase DNA mutation rates in the germline of plants and animals, allowing such variations to be transmitted to subsequent generations. While most mutations are neutral, some may be deleterious, and few can confer adaptive advantages, driving evolutionary changes that enhance survival in urban contexts.

Moreover, urban selective pressures can favor ancestral genetic variants that confer resistance to urban stressors. These variants, already present in populations before urban exposure, may become prevalent under urban conditions. At the same time, cities often present physical barriers, such as buildings and roads, which can reduce population sizes and lead to physical and genetic isolation, thereby limiting gene flow. These phenomena can amplify genetic drift, induce founder effects in newly established urban populations, and create artificial selective pressures, such as those resulting from pesticide use. Such processes contribute to a loss of genetic diversity within populations and significant differentiation among them.

In addition to heritable changes, many adaptations also concern variations in gene expression that guarantee an immediate and flexible stress response and confer phenotypic plasticity (Johnson and Munshi-South 2017). These changes are specific to individuals exposed to stress and are not directly heritable (Lambert and Donihue 2020). However, they may result from epigenetic modifications that alter chromatin accessibility and gene transcription levels. Such changes can be transmitted across multiple generations through transgenerational epigenetic inheritance (TEI) (Fitz-James and Cavalli 2022), allowing biological traits or gene expression patterns to be transmitted without changes in the DNA sequence. Epigenetic marks include DNA methylation, post-translational modifications of histone proteins at N-terminal tails, and the activity of non-coding RNA molecules (Maeji and Nishimura 2018; Lucibelli et al. 2022; Singroha et al. 2022). TEI is frequently induced by persistent environmental stimuli, leading to the fixation of epimutations in populations. Nevertheless, these adaptations are often less stable than genetic changes and may be reversed when the environmental pressures are removed (Lucibelli et al. 2022).

Research on changes in DNA methylation induced by stress conditions on plants suggests that epigenetic mechanisms could play a role in adaptation to urban environments. For instance, studies on *Quercus lobata* have identified associations between DNA methylation profiles and climate gradient (Gugger et al. 2016), showing that plants could adapt to shifts in temperature and climate induced by urbanization. In *Taraxacum officinale* (dandelion), heritable differences in flowering time among apomictic clone members have been linked to DNA methylation patterns, highlighting the contribution of epigenetic variation to heritable phenotypic divergence in ecologically relevant traits (Wilschut et al. 2016).

The evolutionary responses and ecological impacts of urbanization vary depending on city-specific factors, such as size, socioeconomic conditions, and governance (Grimm et al. 2008; Verrelli et al. 2022). Despite these differences, urban areas worldwide share common features, including high population density, extensive impervious surfaces from roads and buildings, elevated temperatures, and significant pollution levels (Oke 1973; Ziter et al. 2019; Miles et al. 2019a). These characteristics make urban environments ideal for studying phenomena such as convergence or parallel evolution, as urban areas are often more environmentally similar to one another than surrounding non-urban regions (Donihue and Lambert 2015). When closely related species are exposed to similar environmental stresses, they usually modify the expression pattern of the same gene families, reflecting repeatability and conservation in the genetic basis of adaptation and evolution. This phenomenon occurs because similar adaptive responses to environmental pressures often involve conserved functional pathways (Mundy 2005; Bohutinska et al. 2021).

In addition, genes with low pleiotropy, those that influence few traits, tend to have high adaptive potential, as mutations in such genes are less likely to produce detrimental pleiotropic effects (Orr 2000). Interestingly, recent studies suggest that, in some cases, highly pleiotropic genes may also play a crucial role in adaptation. Mutations in these genes, which regulate multiple traits, can produce significant phenotypic effects, facilitating fast adaptation and achieving optimal fitness (Whiting et al. 2024).

Adaptation to urban stress is particularly critical for sessile organisms like plants, which cannot move to escape environmental changes (Ashapkin et al. 2020). Consequently, plant adaptability in urban environments represents a fascinating area of study, especially given the diverse contexts in which green spaces are established. While acquiring new phenotypic traits and selecting resistant genetic variants occur over multiple generations, plants rely on rapid modulation of gene expression as an immediate strategy to survive urban stressors (Johnson and Munshi-South 2017).

Plants living in both urban and rural contexts undergo evolutionary changes that influence the introgression of various morphological traits, enabling them to adapt and survive under different conditions. For example, *Digitaria ciliaris*, a common grass found in both settings, exhibits phenotypic variations based on its growth environment. Specifically, plants cultivated in agricultural areas tend to grow faster than those in urban environments, displaying higher height-to-width ratios and thicker stems (Fukano et al. 2020).

This review examines the impact of various biotic and abiotic stresses induced by urbanization on urban vegetation. Defining "urban vegetation" comprehensively is challenging, as each urban area may exhibit unique characteristics linked to the ecological structure of its landscape (Farinati et al. 2022). Although urban areas are often characterized by homogeneity and low biodiversity, urban plant communities can include diverse species (Pearse et al. 2018). The taxonomic composition of urban vegetation varies depending on the type of green space. For instance, in residential neighborhoods, shrubs and cultivated plants are more abundant than large trees, which are more commonly found in courtyards and parks (Threlfall et al. 2016; Pearse et al. 2018). Additionally, urban vegetation may include species of agricultural significance, such as those used for food production and horticulture (Farinati et al. 2022). However, it is important to note that the homogenization of plant communities in urban landscapes is often linked to a reduction in the number of species and overall density (Pearse et al. 2018). Moreover, cities also host both native and non-native plant species, with the latter introduced through human activities such as trade, landscaping, and gardening. Non-native species often exhibit greater adaptability, quickly establishing themselves in urban areas with fewer natural predators and less competition. In contrast, native species may struggle with urban stressors such as pollution and temperature changes. While native plants maintain stronger genetic connections to surrounding natural populations, non-natives may experience limited gene flow (Dylewski Ł et al. 2023).

This review focuses on the genetic adaptations of plants in response to urban challenges, emphasizing the adaptive mechanisms that enable plant survival in such conditions. Understanding these processes is essential for developing strategies to promote urban biodiversity.

## Genetic responses of plants to abiotic stressors

Urban environments exhibit peculiar abiotic characteristics associated with air, soil, and water pollution, rising temperatures caused by heat islands, and light and noise pollution (Theodorou 2022). These environmental changes force plants to adapt to a wide range of adverse conditions. The heterogeneity of the urban areas results in localized microclimates influenced by factors like buildings, roads, pollution, and human activities. These microclimates commonly exhibit varying temperatures, altered humidity levels, elevated CO_2_ concentrations, and modified wind patterns. City plants may develop heat or drought tolerance, altered flowering times, and changes in seed dispersal mechanisms to adapt to fragmented green spaces. Over time, genetic changes can create distinct urban-adapted plant varieties compared to their rural counterparts (Kemppinen et al. 2024).

In urban and peri-urban areas, abiotic stress is a significant limiting factor for plant growth and survival (Kisvarga et al. 2023; Raza et al. 2020). The response to abiotic stress is regulated by multiple genes that affect different pathways (Table 1), making it generally more complex than the response to biotic stress, which is often controlled by a smaller number of genes or even single genes. This genetic complexity poses greater challenges when addressing abiotic stress in plants (Vinocur and Altman 2005).

**Table 1:** Summary of the pathways involved in plant response to abiotic and biotic stress typical of the urban environments

STRESS	Affected pathway
Air Pollution (Abiotic stress)	Cell wall and membrane modification
	Alteration of metabolic processes
	Alteration of photosynthetic rate
	Response to oxidative stress
	Alteration of plant growth
	Alteration of leaf gas exchanges and photochemical processes in photosystem II
	Mitigation of air pollution
High CO_2_ Levels (Abiotic stress)	CO_2_-induced biomass increase
	Increase of photosynthetic activity
	Alteration of nitrate and ammonium assimilation
	Accumulation of carbohydrates and hormonal signalling alteration
	Alteration of iron deficiency response
Heavy Metal Contamination (Abiotic stress)	Hyperaccumulation of heavy metals in the plant tissue caused by the upregulation of genes encoding metal chelators or transporters
	Overexpression of genes encoding plasma membrane cation transporters
	Secretion of organic substances into the rhizosphere that interact with heavy metal ions, transforming them into less harmful forms
	Incorporation of heavy metal ions into cells, allowing for internal detoxification and storage
	Activation of transporter gene families
	Antioxidant defenses and mitigation of oxidative stress
	Alteration of ethylene biosynthesis and leaf biomass
Heat and Drought (Abiotic stress)	Upregulation heat shock proteins that control proper protein folding, assembly, translocation
	Hormonal signalling alteration
	Structural adaptation: increase of transpiration efficiency by modifying stomatal conductance and distribution; modifications of the root system; increase in rolled leaves
	Upregulation of transposable elements
	DNA methylation to silence transposable elements
Salt (Abiotic stress)	Osmotic stress
	Ionic toxicity
	Upregulation of genes encoding glycine/serine-rich proteins (GRPs) and calcium-dependent protein kinases (CDPKs) to reinforce cell walls, counteracting plasmolysis
	Overexpression of genes encoding water channel proteins
	Activation of transcription factor that orchestrate stress-specific gene expression (NAC, CBF3/DREB1A, MYB)
	Hormonal signalling alteration
	Epigenetic modifications: changes in DNA methylation, histone modifications, regulatory mechanisms mediated by non-coding RNAs
Herbivores attack (Biotic stress)	Regulation of glucosinolate metabolism
	Accumulation of phytohormones

In model species such as *Arabidopsis thaliana* and rice, the knowledge regarding genetic response under stress conditions can be extended to urban plants, as these processes are likely conserved across diverse species commonly found in urban landscapes (Farinati et al. 2022). Also the functional validation studies supporting these stress-response pathways have mainly been conducted in model species. Still, they can be extended to a broader range of plants, including those in urban environments.

**Fig. 1:**
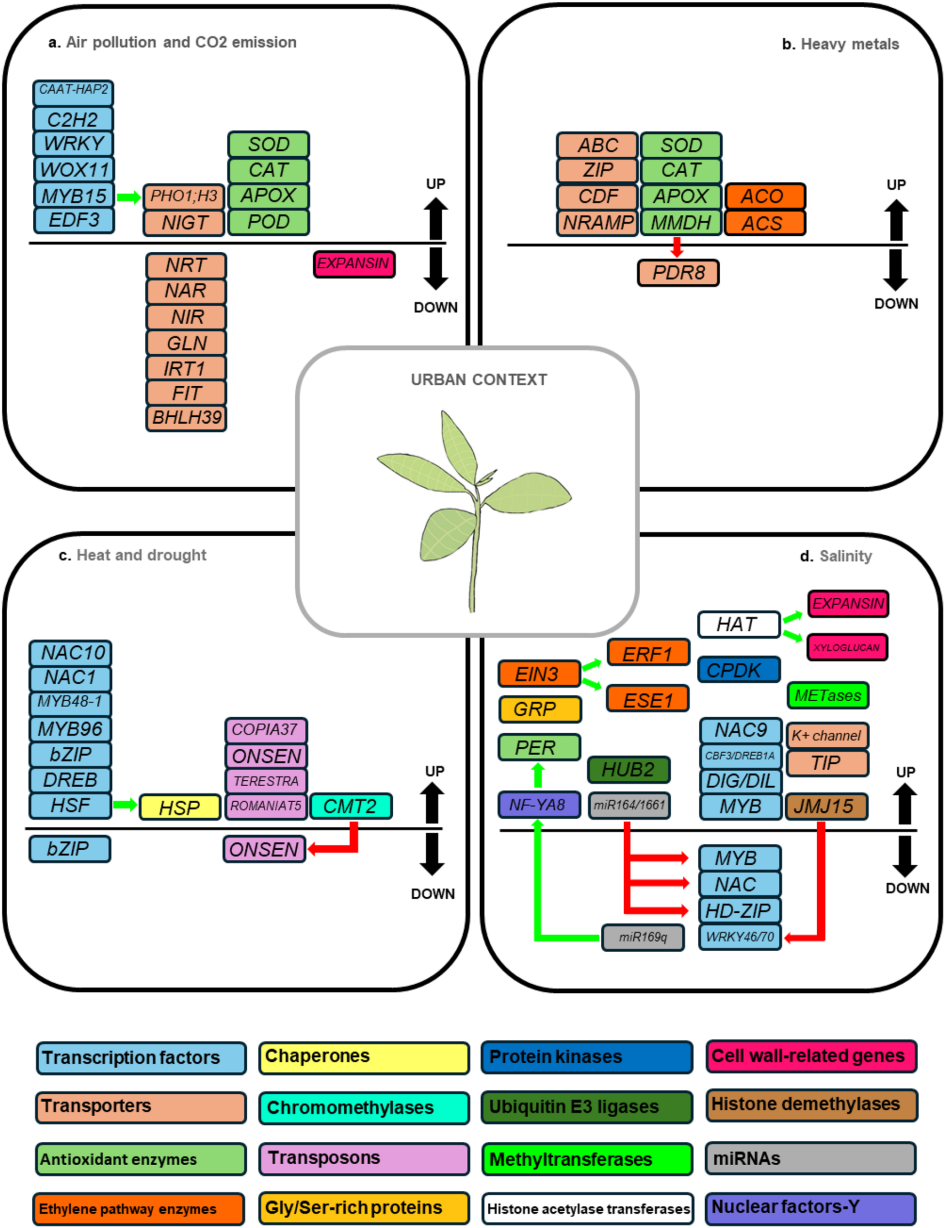
Genetic pathways activated by plants in response to key abiotic stresses in urban environments. The figure illustrates plant molecular responses to air pollution and high CO_2_ emissions (**A**), increased heavy metal levels (**B**), heat and drought stress (**C**), and salinity stress (**D**). Colored boxes highlight the molecular functions of the various genes involved. Genes positioned above the black line are upregulated, whereas those below are downregulated. Green arrows indicate positive regulation, while red arrows indicate negative regulation

This review focuses on some key abiotic stressors, including elevated temperatures, drought, high salinity, heavy metal contamination, air pollution, and increased CO2 levels, significantly affecting plant growth and productivity (Fig. 1).

### Urban vegetation and air pollution

Air pollution and climate change are among the most relevant global environmental challenges. Industrial activities, vehicle emissions, and the combustion of fossil fuels lead to excessive releases of pollutants, including particulate matter (PM), sulfur dioxide (SO_2_), nitrogen oxides (NO_X_), carbon monoxide (CO), and carbon dioxide (CO_2_), the primary greenhouse gas driving global warming. In addition, short-lived air pollutants such as methane, black carbon, and certain hydrofluorocarbons significantly contribute to air contamination (Afifa et al. 2024).

Air pollutants can adversely affect plant growth, resulting in shorter plants with reduced leaf size and fewer leaves. Exposure to PM, in particular, alters the structural composition of leaf wax and modifies the absorption and reflection properties of leaves, ultimately impacting the photosynthetic rate. Moreover, high levels of PM can induce changes in gene expression (Chatterjee et al. 2024). For instance, in *Laurus nobilis* exposed to high-traffic areas, the repression of genes related to the cell wall and membrane modification, such as expansins, has been observed (Vergata et al. 2023b; Chatterjee et al. 2024). Similarly, in *Pithecellobium dulce* exposed to fly ash, an upregulation of antioxidant enzymes, including catalase (CAT), superoxide dismutase (SOD), peroxidase (POD), and ascorbate peroxidase (APX), has been reported (Qadir et al. 2019; Chatterjee et al. 2024) (Fig. 1a).

Transcriptomic analyses on ornamental plants have provided insights into molecular responses to urban pollution. Studies on *Photinia* × *fraseri* and *L. nobilis* in areas with contrasting PM levels revealed significant impairment in the expression of key metabolic genes linked to the tricarboxylic acid (TCA) cycle, gluconeogenesis, and photorespiration. Notably, phosphoglucomutase and sugar isomerase were upregulated only in rural areas, suggesting PM inhibits gluconeogenesis. PM exposure also altered plant defense mechanisms: lower levels upregulated phenylpropanoids and flavonoids, while higher levels induced terpene synthase 14 and suppressed genes involved in cell wall synthesis, indicating structural remodeling. Several transcription factors (WRKYs, C2H2, CAAT-HAP2) were activated under high PM, potentially regulating abiotic stress responses such as proline and soluble sugar accumulation (Vergata et al. 2023a, 2023b).

Additionally, a study on Mediterranean urban plants found that air pollutants impair leaf gas exchange and photosystem II activity, with species-specific differences. *Quercus ilex* exhibited greater resilience than *Nerium oleander* and *Pittosporum tobira*, likely due to its dense trichome coverage, which may mitigate PM-related stomatal dysfunction (Huarancca Reyes et al. 2022).

Despite these challenges, urban vegetation is crucial in mitigating air pollution. Plants absorb CO_2_ and gaseous pollutants through their stomata, while particulate matter is intercepted and retained on leaf surfaces. Species with dense trichomes or waxy coatings on their leaves are particularly effective in capturing airborne particulates (Farinati et al. 2022).

#### ***Adaptation to high CO***_***2***_*** levels***

Fossil fuel combustion is the primary anthropogenic source of atmospheric CO_2_ emissions in the modern era. Urbanization processes significantly influence atmospheric CO_2_ levels. For instance, in urban environments, a 1% increase in population is associated with a 0.95% rise in CO_2_ emissions (Mehmood and Mansoor 2021).

One of the key plant responses to high atmospheric CO_2_ levels is an increase in photosynthetic activity (Poorter et al. 1997; Matros et al. 2006; Penuelas et al. 2013; Singh and Agrawal 2015; Xu et al. 2015b; Drake et al. 1997). Ribulose-1,5-bisphosphate (RuBP) carboxylase/oxygenase (Rubisco) plays a central role in this process by catalyzing the carboxylation of RuBP, enabling CO_2_ fixation. Since the carboxylation reaction of RuBP is not saturated at ambient CO_2_ concentrations, an increase in CO_2_ availability enhances the carboxylation rate, leading to higher photosynthesis rates. This increase drives carbohydrate synthesis and biomass accumulation (Cassan et al. 2023).

Recent studies have identified several transcription factors in *A. thaliana* involved in biomass production under elevated CO_2_ conditions. For example, mutants of the transcription factor genes *MYB15*, *WOX11*, and *EDF3* exhibit significantly reduced biomass growth under high CO_2_, suggesting these factors are crucial for the CO_2_-induced biomass increase. In particular, *MYB15* plays a vital role by regulating the expression of the phosphate transporter genes *PHO1;H3* in roots. High CO_2_-induced plant growth is closely linked to phosphate accumulation, highlighting the role of these transcription factors in the response to elevated CO_2_ (Cassan et al. 2023).

Increased photosynthesis under high atmospheric CO_2_ levels also results in greater production of carbohydrates, primarily sucrose and starch. Sucrose is the main product of photosynthesis, while starch is the plant's central sugar reserve. Under normal CO_2_ conditions, starch accumulates during the day and is consumed at night. However, in high CO_2_ conditions, not all the starch is depleted, leading to its accumulation in the leaves. The excess carbohydrates in leaves are subsequently redistributed to other plant organs, with the distribution pattern varying between species (Thompson et al. 2017).

Since sugars play a role in hormonal regulation pathways, elevated CO_2_ indirectly affects hormonal signaling by altering sugar accumulation (Thompson et al. 2017). For instance, glucose promotes the biosynthesis of auxin, abscisic acid, and ethylene, thereby influencing pathways regulated by these hormones (Lilley et al. 2012; Cheng et al. 2002; Price et al. 2004).

Interestingly, the plant's response to high CO_2_ levels is strongly influenced by its nutritional status. When soil nitrate or iron availability is high, elevated CO_2_ does not significantly alter gene expression in root tissues. However, CO_2_ levels are critical in driving transcriptional changes under nutrient-deficient conditions. In *Arabidopsis*, high CO_2_ has been shown to suppress the expression of genes involved in nitrate and iron deficiency responses, underscoring the importance of nutrient availability in gene regulation (Cassan et al. 2023). Specifically, genes encoding nitrate transporters (*NRT2.1*, *NAR2.1*, and *NRT1.1*) and those involved in nitrate and ammonium assimilation (*NIR1* and *GLN1.2*) exhibit reduced expression under high CO_2_ conditions, particularly in nitrogen-deficient soils. Conversely, genes encoding *NIGT* transcription factors, which repress nitrate transporter gene transcription, are overexpressed. A similar decreased expression pattern is observed for genes involved in the iron deficiency response, such as the transporter *IRT1* and transcription factors *FIT* and *BHLH39* (Cassan et al. 2023) (Fig. 1a).

Significant gene expression changes in response to long-term CO_2_ have been observed also in other plant species. For example, a multigenerational transcriptomic study on *Plantago lanceolata* showed that most transcriptomic responses to high CO_2_ exposure were not stably inherited across generations, indicating that acclimation, rather than genetic adaptation, is the predominant response to rising CO_2_ in this plant species (Watson-Lazowski et al. 2016).

### Genetic mechanisms for tolerance to heavy metal contamination

Heavy metals (HMs) are released into the environment due to rapid urbanization, industrialization, and increased motor vehicle usage (Zhang et al. 2018). HM stress poses a significant abiotic challenge to plants, causing deleterious effects on their growth and development (Sharma 2012; Nagajyoti et al. 2010). Metals such as Zn, Cu, Pb, Mn, Ni, Cr, Cd, and As are persistent and non-biodegradable, mainly when discharged into wastewater, posing serious risks to ecosystems and food chains (Singh et al. 2010). While some metals (e.g., Zn and Cu) are essential for plant growth in trace amounts, others, like As, Hg, Cr, Cd, and Pb, are non-essential and highly toxic even at low concentrations (Li et al. 2013).

Extensive land contamination with HMs is linked to agricultural inputs (e.g., pesticides and fertilizers), municipal waste, compost, and industrial activities such as smelting and mining (Yang et al. 2005). For instance, chemical industry emissions in urban areas contribute to Cd, Zn, and Pb soil contamination [37]. Similarly, heavy metals such as Zn, Cr, Ni, Cd, Cu, and Pb are released during cement production, while volatile Hg accumulates in soils (Engle et al. 2005). In agricultural soils, Cd, As, Pb, Zn, and Cu sources include sewage sludge, pesticides, and fertilizers (Kelly et al. 1996).

Plants have evolved diverse strategies to mitigate HM toxicity, varying by species, HM concentration, and exposure duration (Horst et al. 2010). These strategies fall into two primary mechanisms:

- Exclusion Mechanism: Plants release organic compounds into the rhizosphere to bind HM ions, converting them into less toxic forms and preventing their uptake by roots.
- Detoxification Mechanism: Plants absorb HM ions into their cells, enabling internal detoxification and sequestration (Kochian et al. 2015).

HMs exposure activates various transporter gene families. For example, ZIP transporters facilitate the uptake and transport of metal cations like Zn, Fe, Mn, and Cd in both model and non-model plants (Lin et al. 2009; Zheng et al. 2018; Fan et al. 2021). ABC transporters also play a key role in HM tolerance by removing toxic metals, particularly Cd and Hg (Stein et al. 2006). Cation diffusion facilitators (CDFs) contribute to Zn, Cd, Cu, and Mn detoxification (Fu et al. 2017), while NRAMP (Natural Resistance-Associated Macrophage Protein) transporters are involved in metal uptake, transport, accumulation, and detoxification (Zhang et al. 2020; Tian et al. 2021).

Some plants, the hyperaccumulators, actively absorb high concentrations of HMs and translocate them to aerial tissues. In contrast, the excluder plants minimize HM uptake. The hyperaccumulation of HMs is often linked to the upregulation of genes encoding metal chelators or transporters involved in exclusion and detoxification (Schellingen et al. 2014; Wu et al. 2019; Chen et al. 2019b). For instance, the succulent *Sedum alfredii* is a hyperaccumulator of Cd and Zn (Lu et al. 2013; Tian et al. 2017), with additional tolerance to Pb (Tian et al. 2010) and Cu (Xv et al. 2020). Similarly, studies have demonstrated that the increased Zn uptake in the *Thlaspi caerulescens* and *Arabidopsis halleri* roots is due to the constitutive overexpression of ZIP genes encoding plasma membrane cation transporters (Assunção et al. 2001). Unlike non-hyperaccumulators, hyperaccumulators maintain high ZIP gene expression regardless of Zn availability (Assuncao et al. 2010).

Plants also activate antioxidant defenses in response to Cd and Pb exposure to mitigate oxidative stress caused by reactive oxygen species (ROS). Key antioxidants include glutathione (GSH), ascorbic acid (vitamin C), carotenoids, and tocopherol (vitamin E), distributed throughout plant cells to prevent oxidative damage. Enzymes such as superoxide dismutase (SOD), catalase (CAT), and ascorbate peroxidase (APOX) play critical roles in protecting plant cells from ROS-induced damage (Guan et al. 2009; Pan et al. 2019; Malecka et al. 2021). For instance, plants enhance antioxidant activity under Pb exposure to prevent ROS accumulation. In response to Cd exposure, the mitochondrial malate dehydrogenase (*MMDH2*) gene is upregulated, influencing ROS levels. Overexpression of *MMDH2* leads to H_2_O_2_ accumulation and modulates ROS-mediated signaling, affecting the expression of *PDR8*, a gene encoding a Cd efflux pump, thereby altering Cd tolerance (Wu et al. 2019).

Cd also influences hormone pathways, particularly by impacting ethylene biosynthesis. Cd exposure upregulates genes such as *ACC synthase* (*ACS*) and *ACC oxidase* (*ACO*), which are involved in aminocyclopropane-1-carboxylic acid (ACC) synthesis. Elevated ACC levels promote ethylene production and the expression of ethylene-responsive genes, leading to changes in leaf biomass (Schellingen et al. 2014) (Fig. 1b).

### Coping with heat and drought: genetic and molecular insights

Warm climates often characterize urban areas due to the urban heat island effect caused by impervious surface coverage, anthropogenic heat sources, and reduced plant cover. Excessive heat reduces vapor pressure, increasing atmospheric water demand and lowering soil moisture levels (Farinati et al. 2022). Anthropogenic activities in urban areas exacerbate this issue by increasing air pollutant concentrations, contributing to global warming, and decreasing soil water through evapotranspiration and infrequent rainfall (Manna et al. 2021; Pautasso et al. 2010; Farinati et al. 2022).

Combined heat and drought stress significantly affect plant growth, development, and physiological functions, often occurring concurrently and requiring complex transcriptional regulation mechanisms for drought and thermo-tolerance (Krzyzak et al. 2023; Al-Yasi et al. 2020).

#### Heat shock transcription factors and proteins

Heat shock transcription factors (HSFs) are pivotal in plants' response to heat stress (Kotak et al. 2007). HSFs contain a conserved DNA-binding domain (DBD) with a helix-turn-helix motif and an oligomerization domain (OD) connected by a flexible linker. Based on their OD structures, plant HSFs are classified into three evolutionary classes: A, B, and C (Kotak et al. 2004). HSFs exhibit temporal regulation, with some responding to immediate heat stress and others to prolonged heat exposure and recovery phases (Kotak et al. 2007, 2004; Nover et al. 2001; Lohmann et al. 2004).

Interestingly, HSFs also mediate cross-tolerance between heat and drought stresses. For instance, in *A. thaliana*, the *HsfA3* gene is regulated by DREB2A, a transcription factor involved in dehydration responses (Sakuma et al. 2006). DREB transcription factors bind to the dehydration-responsive element (DRE) motif in the promoters of drought-responsive genes, thereby indirectly enhancing drought tolerance via HSF pathways (Kotak et al. 2007; Yamaguchi-Shinozaki and Shinozaki 1994). During heat stress, HSFs upregulate heat shock proteins (HSPs) (Lindquist 1986). Under normal conditions, HSPs are cytoplasmic but migrate to the nucleus during heat stress. The HSPs (e.g., Hsp110, Hsp90, Hsp70/Hsp80, Hsp60, and small HSPs) act as molecular chaperones, ensuring proper protein folding, assembly, translocation, and quality control (Kotak et al. 2007; Lindquist 1986; Horwitz 1992).

Some HSPs also positively regulate drought tolerance. For example, the *OsHSP50.2* gene in rice, *Oryza sativa*, is upregulated under thermal and osmotic stress, reducing water and electrolyte loss through osmotic adjustment (Xiang et al. 2018).

#### Hormonal and ROS-mediated signal transduction

High temperatures activate additional molecular pathways, including those mediated by hormones and ROS (Kotak et al. 2007). Phytohormones such as abscisic acid (ABA), salicylic acid (SA), and ethylene are central to heat stress responses. Elevated levels of these hormones have been observed under heat shock, and mutants in ABA and ethylene signaling pathways exhibit alteration of heat sensitivity and drought tolerance (Kotak et al. 2007; Larkindale et al. 2005b, 2005a; Larkindale and Huang 2004; Lee and Luan 2012).

Transcription factors (TFs) involved in ABA-dependent pathways include the basic leucine zipper (bZIP) family, which positively regulates ABA-responsive genes in *A. thaliana* and is implicated in osmotic stress responses in rice and other plants (Kotak et al. 2007; Choi et al. 2000; Saxena et al. 2013; Joo et al. 2019b; Yoon et al. 2017; Zou et al. 2008). In particular, functional analyses have demonstrated the role of ABA-dependent pathways in drought tolerance. For instance, overexpression of the *Arabidopsis ABF2* gene (containing a bZIP domain) in transgenic cotton enhances drought resistance by activating ABA-dependent genes (Liang et al. 2016). Conversely, silencing the *ATBZ1* gene in pepper, which also contains a bZIP domain, improves drought tolerance, highlighting the dual regulatory role of bZIP TFs (Joo et al. 2019a).

MYB TFs also contribute to ABA-mediated drought tolerance. In *Arabidopsis*, the *AtMYB96* gene integrates ABA and auxin signaling to enhance drought resistance (Lata and Prasad 2011; Seo et al. 2009). In rice, upregulation of *OsMYB48-1* activates multiple ABA-related genes, promoting drought tolerance (Xiong et al. 2014).

#### NAC transcription factors and structural adaptations

Plants can adopt various physiological strategies to cope with stressful conditions, resulting in significant structural changes that help them adapt to the environment. Under water deficit, plants increase transpiration efficiency by modifying stomatal conductance and distribution. In particular, the plant implements various modifications to the root system, which tends to develop deeper and extended roots. Furthermore, relatively more significant growth of roots compared to shoots is observed in plants subjected to drought stress. At the leaf level, an increase in rolled leaves is observed. As a result of these structural adaptations, plants can maintain physiological function and increase their chances of surviving in stressful environments (Seleiman et al. 2021). These structural changes are related to specific TFs that activate distinct molecular pathways. Different TF families act synergistically, orchestrating multi-faceted responses to simultaneous heat and drought stress (Manna et al. 2021).

The NAC TF family plays a crucial role in structural adaptations to drought. In *O. sativa*, specific NAC genes regulate root structure to optimize water uptake under drought conditions (Hu et al. 2006; Nakashima et al. 2007; Redillas et al. 2012; Tran et al. 2004). For example, *OsNAC10* is a key player in modifying root architecture. Overexpression of *OsNAC10* in rice leads to root enlargement and improved drought tolerance in transgenic plants. Additionally, target genes of OsNAC10 have also been identified, including stress-response genes such as the potassium transporter *HAK5*, protein kinases, and various TFs (Jeong et al. 2010). Furthermore, *OsNAC1* has been shown to play a role in stomatal closure. Its activity in regulating stomatal opening under water stress was demonstrated through functional analysis, observing active transcription of the GFP reporter gene driven by the *OsNAC1* promoter in stomatal guard cells, the kidney-shaped cells responsible for controlling stomatal opening (Hu et al. 2006).

#### Genomic stability and transposable elements

High urban temperatures exert significant stress on plants and can destabilize their genetic material by upregulating the mobilization of transposable elements (TEs) (Sun et al. 2020). TEs are segments of DNA capable of moving and inserting themselves into different genomic locations. Depending on their insertion points, TEs can induce various effects, such as disrupting gene function or regulating gene expression. The mobilization of TEs under high-temperatures conditions is particularly intriguing, as it may contribute to stress adaptation by activating new genetic regulatory pathways (Sun et al. 2020; Roquis et al. 2021).

In *A. lyrata* and *A. thaliana*, several families of heat-sensitive TEs, including *ONSEN*, *COPIA37*, *TERESTRA*, and *ROMANIAT5*, have been identified. The heat reactivity of these transposons is attributed to the presence of heat-responsive elements (HREs) in their sequences (Pietzenuk et al. 2016). Some TEs exhibit preferential insertion sites; for instance, *ONSEN* tends to insert into regions enriched with the histone variant H2A.Z and H3K27me3. Notably, under high-temperature conditions in *Arabidopsis*, 61 genes that are normally unresponsive to stress become activated following the insertion of *ONSEN*. These insertion events often occur in genes associated with abiotic stress response, such as those involved in the phosphatidylinositol signaling system and NAD + biosynthesis, highlighting the potential adaptive role of TEs (Roquis et al. 2021).

However, TE mobilization can frequently lead to mutations and genomic instability with harmful consequences for plants. Transposon activity can disrupt gene function, cause gene silencing, and fragment DNA, ultimately impairing cellular functions. During thermal stress, uncontrolled TE mobilization can trigger a cascade of events that compromise genomic stability and alter phenotypic traits.

To mitigate these deleterious effects, plants employ DNA methylation to silence TEs. For example, chromomethylase 2 (CMT2) suppresses the heat-stress-induced retrotransposon *ONSEN* in *Arabidopsis*. Disruption of methylation pathways leads to increased TE activity, which can have severe genetic consequences (Nozawa et al. 2022) (Fig. 1c).

### Salt stress responses: genetic pathways and adaptations

The increase in soil salinization is driven by various factors associated with anthropogenic activity and urban pollution. Climate change exacerbates this issue with reduced rainfalls, frequent extreme weather events, and rising sea levels. Additionally, urbanization and industrial waste have contaminated aquifers, leading to the accumulation of salt deposits in the soil (Litalien and Zeeb 2020). Soil salinity is one of the most severe abiotic stresses in agriculture, significantly limiting plant growth and reducing crop yields, posing a critical threat to global food security (Singh and Roychoudhury 2021). Beyond agriculture, salinity impacts ecosystems by reducing plant diversity and richness, which reduces nutrient sources for other organisms, thereby disrupting ecosystem balance and biodiversity (Kefford et al. 2011; Dowse et al. 2017; East et al. 2017). Reduced plant growth also results in a decline in organic carbon in the soil, a major carbon source, further impacting ecosystem functions (Setia et al. 2013).

#### Physiological impact of salt stress

Salt stress affects plants through two primary mechanisms: osmotic stress and ionic toxicity (Shavrukov 2013). Osmotic stress occurs immediately upon salt exposure, disrupting the osmotic gradient essential for water uptake by roots. In saline soils, water availability to plants decreases, leading to cellular dehydration and misfolding of proteins (Deinlein et al. 2014; Flowers et al. 2015). Reduced cellular water content also decreases vacuolar volume, compromising turgor pressure and causing cell collapse (Litalien and Zeeb 2020).

Ionic stress represents the secondary phase of salt stress. Excess salts, especially sodium and chloride, accumulate within cells. Sodium (Na⁺) ions can interfere with enzymatic activities due to their poor compatibility with proteins. In contrast, chloride (Cl⁻) ions, essential as cofactors in chlorophyll, can cause chlorosis when present in excess, disrupting photosynthesis (Pardo and Quintero 2002; Flowers et al. 2015; White and Broadley 2001). Furthermore, nonspecific ion transporters may fail to distinguish between potassium (K⁺) and sodium, leading to high intracellular Na⁺ levels and subsequent toxicity (Pardo and Quintero 2002).

#### Genetic and molecular responses to salt stress

Plants respond to salt stress by activating distinct genetic pathways that mitigate its effects on cellular functions. The rapid response to osmotic stress occurs within minutes of salt exposure. Key genes involved in this plant response include those encoding water channel proteins, such as tonoplast intrinsic proteins (TIPs), which regulate water balance between intra- and extracellular environments. Additionally, potassium channel proteins act as ion antiporters, reducing sodium toxicity (Shavrukov 2013; Ayarpadikannan et al. 2012).

Early osmotic stress responses in rice involve the upregulation of genes encoding glycine/serine-rich proteins (GRPs) and calcium-dependent protein kinases (CDPKs). These proteins reinforce cell walls, counteracting plasmolysis. Notably, GRP and CDPK expression peaks within 15 min of salt exposure, remains elevated for one hour and returns to baseline after three hours (Shavrukov 2013; Kawasaki et al. 2001).

The response to ionic toxicity occurs later and involves the activation of TFs that orchestrate stress-specific gene expression. For example, CBF3/DREB1A TFs confer salt resistance when overexpressed and induce salt hypersensitivity when deleted in *Arabidopsis* (Zhao et al. 2016). NAC TFs also play key roles; for example, the *TaNAC29* gene of *Triticum aestivum* (wheat) enhances salt resistance. The role of *TaNAC29* was demonstrated by functional analysis, obtaining transgenic *Arabidopsis* plants that overexpress *TaNAC29*. Under salt stress, these mutants exhibit lower ROS accumulation, greener leaves, and more stable cell membranes (Xu et al. 2015a).

MYB TFs have been implicated in salt stress responses, as demonstrated in the evergreen *Casuarina equisetifolia*, where seven MYB genes (*CeqMYB164*, *CeqMYB4*, *CeqMYB53*, *CeqMYB32*, *CeqMYB114*, *CeqMYB71*, and *CeqMYB177*) were linked to salt tolerance. The presence of auxin-responsive elements in the promoters of some *CeqMYB* genes suggests that MYB-mediated salt tolerance may involve hormone signaling pathways (Wang et al. 2021). ABA-responsive TFs, such as DIG/DIL, also play critical roles, with DIG overexpression increasing sensitivity to high salt levels (Song et al. 2016). Similarly, EIN3, a master regulator of the ethylene signaling pathway, modulates TFs like ERF1 and ESE1 to enhance salt tolerance (Singh and Roychoudhury 2021).

#### Epigenetic regulation of salt stress responses

Epigenetic modifications are powerful mechanisms that regulate gene expression and enable plants to respond rapidly to salt stress. Salt-induced epigenetic processes in plants include DNA methylation, histone modifications, and regulatory mechanisms mediated by non-coding RNAs (ncRNAs). These processes influence transcriptional activity by altering chromatin structure, thereby modulating the plant's response to environmental challenges (Singroha et al. 2022).

Changes in DNA methylation represent a key adaptive strategy for coping with salt stress. For instance, in *Pyrus betulaefolia*, salt stress leads to an upregulation of methyltransferases (MTases) and salt-responsive genes (Zhang et al. 2023). In *Glycine max* roots exposed to high salt concentrations, methylation profiles are significantly altered across all sequence contexts subject to methylation (CG, CHG, CHH, where H represents any nucleotide other than G) (Chen et al. 2019c). Salt-tolerant plants exhibit increased DNA methylation under saline conditions, while salt-sensitive plants show reduced methylation levels (Ashapkin et al. 2020). Furthermore, hypermethylation induced by high salt concentrations targets cytosine residues within TEs, silencing them and thereby maintaining genomic stability under stress conditions (Lin et al. 2022).

Histone modifications also play a pivotal role in salt stress responses. For example, mono-ubiquitination of the H2B histone subunit by the ubiquitin E3 ligase HUB2 enhances salt tolerance in *Arabidopsis* (Chen et al. 2019a). Similarly, in *Zea mays*, increased expression of histone acetyltransferases ZmHATB and ZmGCN5 correlates with enhanced salt stress resilience. These enzymes acetylate the promoters of cell wall-related genes, such as *ZmEXPANSIN B2* and *ZmXYLOGLUCAN endotransglucosylase/hydrolase1*, thereby upregulating their expression to mitigate osmotic stress (Li et al. 2014). In *Nicotiana tabacum* and *Z. mays*, histone acetylation relaxes chromatin and activates the transcription of stress-response genes, including those encoding peroxidases and antioxidative enzymes. This activation reduces ROS levels and promotes the accumulation of osmotic metabolites, fostering salt tolerance (Li et al. 2014; Singroha et al. 2022; Sokol et al. 2007).

Histone methylation further modulates salt stress regulation by regulating gene activity. For instance, in *Arabidopsis*, the JMJ15 histone demethylase removes H3K4me3 marks from the promoters and coding regions of *WRKY46* and *WRKY70*, genes with negative roles in salt tolerance. This demethylation represses these genes, enhancing plant resistance to saline conditions (Shen et al. 2022).

Finally, salt stress influences the transcription of specific ncRNAs, which modulate gene expression by silencing target genes involved in complex regulatory pathways. In *Z. mays*, salt stress-induced ROS accumulation inhibits the expression of pre-miR169q, leading to the upregulation of its target, the nuclear factor-Y ZmNF-YA8. This transcriptional regulator promotes the expression of the antioxidant enzyme ZmPEROXIDASE1, reducing oxidative stress (Xing et al. 2022). Moreover, many miRNAs act by silencing transcription factors implicated in salt stress responses. For example, miR164 and miR166 in *Z. mays* target MYB, NAC, and HD-ZIP genes, thereby fine-tuning the plant's adaptive response (Singroha et al. 2022; Ding et al. 2009) (Fig. 1d).

## Genetic responses of plants to biotic stressors

In urban ecosystems, ecological niches are altered by the presence of new pathogens, pests, insects, and invasive species (McKinney 2008; Kisvarga et al. 2023). To defend against pathogen infections, plants activate various immune responses, e.g., lignin deposition, cell wall strengthening, and the production of endogenous substances such ROS, SA, and oligosaccharides. These substances help to trigger systemic responses to biotic stresses (Peng et al. 2025).

**Fig. 2:**
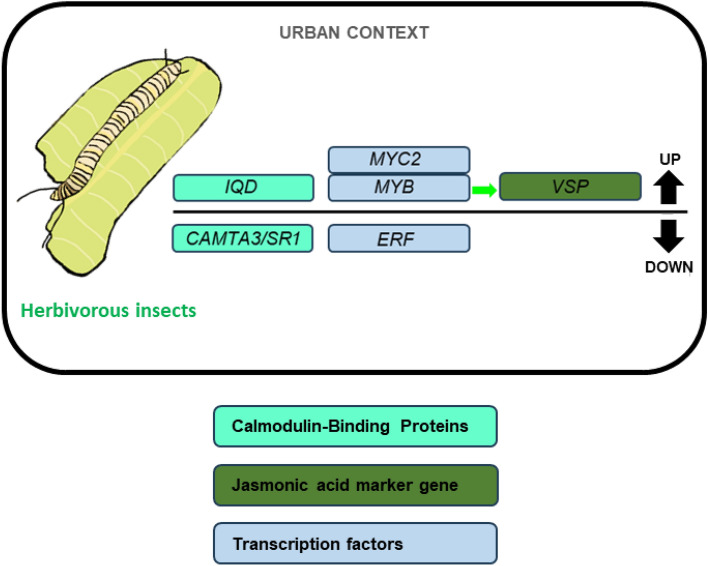
Genetic pathways activated by plants in response to key biotic stressors. This figure illustrates the genetic pathways plants activate as a defense mechanism against herbivorous attacks in urban environments. Colored boxes highlight the molecular functions of the various genes involved. Genes above the black line are upregulated, whereas those below are downregulated. The green arrow indicates positive regulation

In addition to the presence of new pathogens that proliferate due to urban environmental alteration, one of the most significant biotic stresses for plants in urban environments is represented by arthropod herbivore attacks (Fig. 2). Urbanization-induced ecosystem alterations pose novel challenges for herbivorous arthropods, their predators, and host plants, influencing ecology and evolution in urban and surrounding areas (Anjali et al. 2023; Miles et al. 2019a).

The urban heat island effect modifies thermal conditions and affects the breeding seasons of herbivorous arthropods and their predators (Imhoff et al. 2010). Urban light and chemical pollution also represent stress for urban-dwelling organisms (Isaksson 2015). In addition, habitat fragmentation due to urbanization isolates organisms, leading to population declines (Grimm et al. 2008). These declines disrupt trophic dynamics within plant–herbivore communities, affecting bottom-up and top-down processes (Hope et al. 2003; McKinney 2008; Aronson et al. 2014). Understanding pathogen ecology in urban environments is critical for mitigating disease risks to wildlife and, in some cases, humans. Many pathogens can infect multiple host species, threatening human health and vulnerable wildlife populations (Cleaveland et al. 2001; Woolhouse et al. 2001; Woolhouse and Gowtage-Sequeria 2005).

### Genetic strategies for defense against herbivores

Insect herbivores are more prevalent in the hottest parts of cities due to favorable conditions created by higher temperatures and urban heat islands (Youngsteadt et al. 2015; Meineke et al. 2013; Dale and Frank 2014). In addition, there is a correlation between insect herbivore abundance and plant water stress, as herbivory, particularly sap-feeding insects, exacerbates water stress (Cockfield and Potter 1986; Kansman et al. 2022).

Plants employ diverse defense mechanisms against biotic threats, including genetic response, physical barriers such as hairs, trichomes, spines, thorns, and cuticles, as well as chemical strategies like the accumulation of secondary metabolites (SMs), including volatile organic compounds (VOCs) (Lewandowska et al. 2020; Wang et al. 2020; Strauss and Irwin 2004) (Table 1).

Genes involved in plant secondary metabolism and hormonal signaling change their expression level under herbivory stress through epigenetic modifications. Research on *Arabidopsis* has demonstrated that histone modifications, DNA methylation, and RNA-directed DNA methylation (RdDM) are associated with the expression of chemical defenses, such as glucosinolates and flavonoids. Additionally, studies on both invasive (*Ambrosia artemisiifolia*, *Solidago canadensis*) and non-invasive species (*Viola cazorlensis*) have associated epigenetic variation with responses to herbivory and invasive potential (Mounger et al. 2021).

One chemical defense is the glucosinolate-myrosinase system, common in the Brassicaceae family, including crops and the model plant *A. thaliana* (Fahey et al. 2001; Kliebenstein 2008; Textor et al. 2004; Wittstock and Halkier 2002). When plant tissue is damaged, myrosinases hydrolyze glucosinolates (GSLs) into glucose and unstable intermediates that rearrange into reactive products (Kliebenstein 2008; Wittstock and Halkier 2002), contributing to defense against pathogens and herbivores (Kliebenstein et al. 2002; Lambrix et al. 2001; Tierens et al. 2001).

Genes from the IQ-Domain family are involved in glucosinolate synthesis and other processes, such as fruit development and cell shape formation (Zentella et al. 2007; Xiao et al. 2008; Burstenbinder et al. 2013, 2017; Guo et al. 2021; Bao et al. 2021). These genes have been identified in various plant species, including rice, tomato, soybean, grapevine, and *Arabidopsis*. In *A. thaliana*, 33 *IQD* genes encode proteins with a conserved plant-specific IQ67 domain characterized by calmodulin recruitment motifs. The *IQD1* gene encodes for a nuclear-localized protein that binds calmodulin in a calcium-dependent manner, positively regulating glucosinolate accumulation. Overexpression of *IQD1* enhances resistance against herbivores such as *Trichoplusia ni* and *Myzus persicae*, suggesting its role in perceiving calcium signals to regulate plant defenses (Abel et al. 2005; Levy et al. 2005).

Another regulator, the *Arabidopsis* Ca2 + /calmodulin-binding transcription factor AtSR1/CAMTA3, suppresses defense responses against biotrophic pathogens and herbivores. CAMTA3 also controls glucosinolate metabolism, further linking calcium signaling with plant defense (Laluk et al. 2012; Qiu et al. 2012; Du et al. 2009; Galon et al. 2008).

TFs are pivotal regulators of plant secondary metabolism, functioning either as molecular on/off switches (type I TFs) or as modulators that operate under specific environmental or physiological conditions (type II TFs) (Chen et al. 2024). Among these, the MYB family plays a central role in key biological processes, including growth, reproduction, and stress responses.

Within the Brassicales order, MYB28 and MYB29 were among the first type I TFs identified for their regulatory role in secondary metabolism (Sonderby et al. 2007; Beekwilder et al. 2008). MYB28, in particular, serves as a crucial integrator of carbohydrate availability and external environmental cues, orchestrating the transcriptional activation required for the biosynthesis of aliphatic glucosinolates. These secondary metabolites enhance plant resistance to herbivores by serving as chemical deterrents (Gigolashvili et al. 2007b).

For indolic glucosinolate biosynthesis, MYB34, MYB51, and MYB122 have been identified as key regulators. MYB51 predominantly controls biosynthesis in the shoots, whereas MYB34 is more active in the roots, indicating a spatial specialization in their regulatory roles. MYB122 has a complementary role, particularly under environmental stress conditions, enhancing the plant's adaptive metabolic responses (Celenza et al. 2005; Gigolashvili et al. 2007a; Malitsky et al. 2008; Frerigmann and Gigolashvili 2014).

This function partition among MYB transcription factors highlights their sophisticated regulation of secondary metabolism, enabling plants to fine-tune their biochemical defenses in response to developmental and environmental challenges.

In response to herbivory, plants detect herbivore-associated molecular patterns (HAMPs) and damage-associated molecular patterns (DAMPs) (Acevedo et al. 2015). These signals trigger the accumulation of phytohormones such as jasmonic acid (JA), ABA, and ethylene, which initiate defense signaling cascades. JA, particularly its active isoleucine conjugate (JA-Ile), activates transcriptional responses to wounding stress (Howe and Schaller 2008; Wasternack and Hause 2013). The JA signaling pathway consists of MYC and ERF branches, responding to herbivory and pathogen attacks. Chewing herbivores activate the MYC branch, controlled by MYC2, MYC3, and MYC4, which regulate JA-responsive genes like *VEGETATIVE STORAGE PROTEIN 1* and *2* (Verhage et al. 2011; Vos et al. 2013). Conversely, the ERF branch is active during necrotrophic pathogen infection. The MYC and ERF branches antagonize each other; herbivores such as *Pieris rapae* prefer plants with ERF-branch activation. Activating the MYC2 branch reduces herbivore attraction, enhancing plant defenses (Anderson et al. 2004; Lorenzo et al. 2004; Fernandez-Calvo et al. 2011; Niu et al. 2011) (Fig. 2).

## Genotypic adaptations of plants to urban environments

Urbanization imposes unique challenges on plant populations, resulting in genetic adaptations that enhance survival in these altered ecosystems. The stresses of urban environments, ranging from pollution and soil compaction to water scarcity and elevated temperatures, necessitate significant evolutionary responses. Understanding these genetic traits is critical, as they provide valuable insights into stress tolerance mechanisms. Furthermore, the introgression of these traits into less resilient plants allows the development of cultivars better suited to urban conditions (Farinati et al. 2022).

One striking example of urban genotypic adaptation is observed in white clover (*Trifolium repens*), where the frequency of hydrogen cyanide (HCN) production, a chemical defense against herbivores, declines in urban populations. In North American urban white clover populations, there is a more pronounced reduction in HCN production in warmer areas than in colder ones, suggesting temperature-dependent selective pressure favoring non-HCN-producing genotypes in urban habitats (Thompson et al. 2016; Santangelo et al. 2020). The Global Urban Evolution Project applied an integrated genomic and phenomic approach to investigate adaptation in *T. repens*, combining large-scale population genomic analyses with phenotypic assessments of cyanogenesis across urban and rural environments. Researchers analyzed over 110,000 samples from 6,169 populations across 160 cities worldwide, revealing a consistent reduction in HCN production in plants of urban environments. This pattern suggests that urban factors, such as reduced herbivory pressure and milder temperatures, select against cyanogenesis. These results demonstrate that urbanization drives parallel evolutionary adaptations in *T. repens* populations (Santangelo et al. 2022).

Concerning divergent evolutionary responses in plant–herbivore interactions, *A. thaliana* populations in urban areas experience higher aphid densities but do not evolve increased resistance or tolerance to aphid herbivory. However, urban plants show enhanced tolerance to caterpillar herbivory under controlled conditions. This suggests that urban environments shape herbivore-specific plant responses, promoting adaptations specific to particular herbivore pressures (Miles et al. 2019a; Qu et al. 2022).

Reproductive success and mating systems, both vital for species persistence, are also significantly influenced by urbanization. For example, *Paubrasilia echinata*, an endangered species, has lower reproductive success in urban environments than Atlantic forest populations. Urban plants of this species produce fewer flowers, have reduced fruit sets, and exhibit lower seed germination rates, likely due to stressors such as pollution and habitat degradation (Oliveira et al. 2019). Additionally, urbanization and habitat fragmentation have been associated with shifts in plant mating systems. In fragmented environments, reduced pollinator availability often drives a transition from cross-fertilization to self-fertilization. While self-fertilization offers short-term reproductive assurance in isolated populations, it reduces genetic diversity and long-term adaptability to changing environments (Eckert et al. 2010).

Research on *Brassica incana*, a Mediterranean cliff species, further reveals the impact of urbanization on reproductive success and phenotypic traits. Urban populations exhibit reduced connectivity between plant groups, leading to decreased genetic exchange and lower reproductive success. These populations produce fewer flowers and display decreased seed viability than those in natural habitats. Such reduction confirms the trend observed across urban environments: reduced genetic diversity, changes in reproductive strategies, and compromised adaptability to future environmental pressures (Laccetti et al. 2025).

Together, these findings illustrate the deep influence of urbanization on plant genotypes, reproductive strategies, and ecological interactions, highlighting the complex interplay between environmental pressures and evolutionary processes.

## Conclusions and future perspectives

Deciphering the molecular mechanisms underlying plant responses to the complex stress factors associated with urbanization remains a significant challenge. Recent advances in genome-editing technologies and multi-omics approaches offer a powerful framework for addressing this challenge by enabling the analysis of genetic changes, identification of specific molecular biomarkers, monitoring of plant physiological states, and prediction of plant responses to diverse stressors (Raza et al. 2020; Naeher et al. 2022; Yin et al. 2024). CRISPR/Cas9 technology, widely used for crop improvement, could also facilitate the identification of genes involved in urban adaptation and support plant restoration in cities facing rising temperatures, elevated CO_2_ levels, and drought (Erdoğan et al. 2023; Yin et al. 2024). These strategies could enhance plant resilience to anthropogenic impacts on natural ecosystems (Dempewolf et al. 2017; Sharma et al. 2021).

Artificial intelligence (AI), particularly machine learning, could help predict plant stress responses and optimize urban green space design (Ali et al. 2024). AI-driven models can support selecting plant species suited for urban conditions, considering climate, soil, and aesthetic factors. Additionally, machine learning can optimize green space placement to mitigate urban heat islands, improve air quality, and regulate temperature (Liu 2022; Ahn et al. 2025). Future research should integrate genetic data into these models, refining predictions of urbanization’s impact on plant adaptation (Toro et al. 2024).

Identifying adaptation markers plays a central role in conserving biodiversity within human-altered landscapes. By enhancing our understanding of these markers, we can devise strategies to maintain genetic diversity and improve the resilience of wild plant populations. Such efforts are essential for safeguarding plant species against the growing environmental pressures of urbanization, ensuring their persistence in increasingly challenging ecosystems (Dearborn and Kark 2010).

Combining multi-omics, genome editing, and AI with ecological and evolutionary studies will provide a comprehensive framework for understanding plant adaptation in urban environments. This integrated approach will enhance biodiversity conservation efforts and the development of resilient plant populations in response to global change.

## Data Availability

The data presented in this study are available in the manuscript.
